# Securing Data in Vehicles: Privacy-Preserving Frameworks for Dynamic CAV Environments

**DOI:** 10.3390/s26041326

**Published:** 2026-02-19

**Authors:** Rahma Hammedi, David J. Brown, Omprakash Kaiwartya, Pramod Gaur

**Affiliations:** Department of Computer Science, Nottingham Trent University, Nottingham NG11 8NS, UK; rahma.hammedi@ntu.ac.uk (R.H.); david.brown@ntu.ac.uk (D.J.B.); pramod.gaur@ntu.ac.uk (P.G.)

**Keywords:** CAVs, privacy, federated learning, permissioned blockchain, software defined vehicular networking

## Abstract

Advancements in the Connected and Autonomous Vehicles (CAVs) industry are revolutionizing modern transportation through advanced automation levels and connectivity capabilities. While autonomous vehicles can operate using onboard sensors alone, the integration of Vehicle-to-Everything (V2X) communication is vital for enabling seamless connectivity and cooperative decision-making. However, the increasing exchange of traffic and sensor data introduces critical privacy challenges, necessitating robust and scalable privacy-preserving mechanisms to ensure user trust and compliance with data protection regulations. The inherently dynamic nature of CAV environments, characterized by high mobility, short-duration connections, and frequent handovers, further complicates the design of effective privacy models. In this context, this paper investigates the evolving data privacy risks associated with CAV systems. It critically reviews existing privacy-preserving approaches and identifies their limitations in dynamic vehicular contexts. In particular, the paper explores the role of Federated Learning, permissioned blockchain and Software-Defined Networking (SDN) as enabling technologies for privacy preservation in CAVs. The analysis concludes with targeted recommendations for optimizing these frameworks to enhance privacy resilience in next-generation intelligent transportation systems.

## 1. Introduction

The transportation sector is undergoing a significant transformation driven by the emergence of Connected and Autonomous Vehicles (CAVs) [1,2,3] (see Table 1). The advances in CAVs depend primarily on the integration of advanced driver-assist systems (ADAS). ADAS, a set of vehicular systems and technologies, is used to transform human-driven vehicles into a fully driverless option [4]. It uses various onboard sensors to collect and exchange data about the vehicle’s surroundings and provide feedback or take needed actions without any human intervention. This traffic data collection process is designed using a wireless communication technology, known as Vehicle-to-Everything, leading to a huge amount of data being wirelessly transmitted [5]. This basically opens potential privacy vulnerabilities for hackers to access the in-vehicle network. A hacker or any unauthorized node may have ways of accessing data, corrupting systems, manipulating records, and tracking every vehicle movement. Recent research undertaken by independent research organizations from EUROPE/US [6] showed that once an adversary can access the in-vehicle network, they have the potential to manage all internal processes of a vehicle. This is from manipulating the vehicle’s acceleration, to applying/releasing brakes, locking/unlocking the doors, poisoning ECUs through injecting malicious software, and is even capable of downloading inaccurate or tampered geographic information. It could not only compromise the functionality of the vehicle but also potentially access or alter personal data stored within the vehicle’s systems.

Over the last few years, various studies have attempted to investigate emergent solutions to cope with privacy violations in CAVs. Driven by the need to obtain insights into data generated from different vehicles, Artificial Intelligence disciplines, e.g., machine learning (ML), have been extensively deployed to protect drivers’ information [7]. However, ML requires data collected by vehicles to be transmitted and handled centrally within a cloud-based server or data center. Such a cloud-centric approach causes unacceptable latency and communication inefficiency [8]. To address these issues, a decentralized ML approach named Federated Learning (FL) has been recently introduced [9]. FL guarantees that the raw data never leaves the vehicle. Instead, vehicles send only model parameters to the central server for aggregation and management [10], which maintains the privacy of the vehicle’s local data and decreases the transmission overhead. Nevertheless, even FL is well-suited to operate with distributed training nodes, it relies on a single central server, which poses serious issues, such as a single point of failure, i.e., overhead in the central entity, since all FL network data is aggregated on one server [11]. To address this, blockchain is proposed to offer a fully immutable and decentralized architecture using several miners [12]. Current solutions show that integration of blockchain with Federated Learning is an efficient strategy to ensure secure and intelligent data sharing. Moving from a network legacy architecture with one central server to a decentralized network improves the overall system performance by enabling reliable, low-latency connectivity and global consciousness throughout the whole network. However, due to the huge data quantity delivered by vehicles, servers have inherited numerous issues including storage limitations [10]. There will be an overload as long as more transactions are added over time [13], which potentially leads to scalability issues and increases storage requirements, especially for nodes that maintain the entire blockchain log. Hence, a cloud storage network has arisen [14]. Software-Defined Vehicular Networking (SDVN) is a prospective solution to solve any storage, management, and data protection-related issues. The main concept of SDVN is to decouple the data plan from the control plan, and to offer a fully programmable software-driven network [12]. The control plan is a virtualized entity designed to control the whole network, while the rest of the network’s entities are only left for data forwarding.

**Figure 1:**
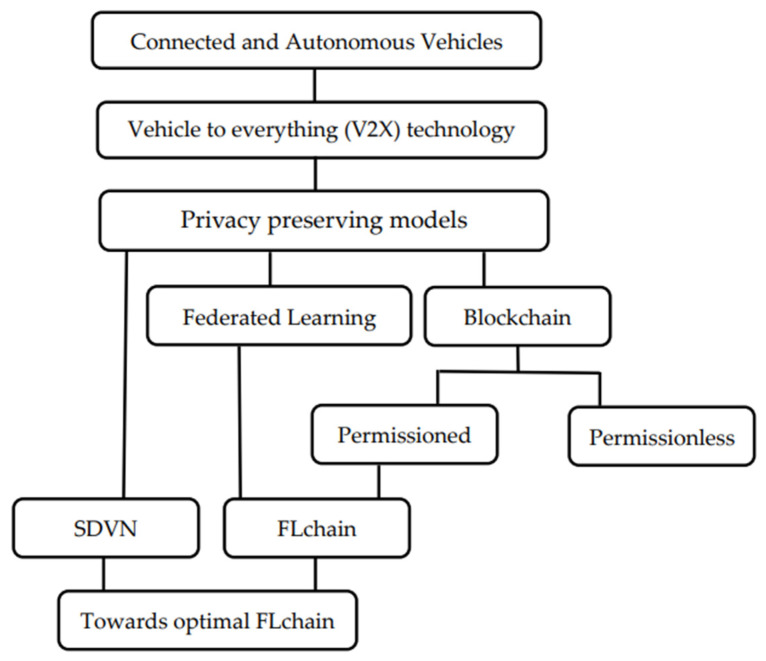
Organization of the paper.

As illustrated in Figure 1, which outlines the organization of the paper, this article firstly explores the Connected and Autonomous Vehicles infrastructure, including their specifications and the used technologies. In Section 2, we elaborate on relevant studies concerning privacy-preserving solutions in CAVs infrastructure, such as Federated Learning, blockchain, and the combination of both technologies. As this combination, which will be named FLchain, requires further privacy enhancements under certain conditions, researchers suggested utilizing a permissioned blockchain instead of the classical blockchain to add more privacy barriers to the network. The detailed description of this framework is presented in Section 3. Then, in Section 4, we delve into the Software-Defined Vehicular Networking and review literature on existing privacy threats in this architecture. Finally, we explore potential improvements toward achieving an optimal privacy-preserving schema in CAVs, offering enhanced features in terms of latency, coverage, and computation.

## 2. Connected and Autonomous Vehicles (CAVs)

Vehicular Networks [13] are wireless communication systems in which vehicles are equipped with radio interfaces enabling data exchange among each other, with the cellular network, and with stationary infrastructure components such as base stations, roadside units, traffic signs, and radar systems [14]. The evolution of conventional vehicles into Connected and Autonomous Vehicles (CAVs) is achieved through the integration of numerous engine control units (ECUs). ECUs manage actuation systems within an internal combustion engine, leveraging data from a range of associated sensors including radar, lidar, internal and external cameras, ultrasound sensors, and GPS [15] (see Table 2). Interpreted data help to adjust the engine actuators and monitor several systems within the vehicle accordingly, ranging from the entertainment system to vehicle control.

However, collected data are inherently heterogeneous due to spatial, temporal, and contextual diversity, leading to severe non-Independent and Identically Distributed (non-IID) data propagations that degrade global model convergence and stability [16]. This issue is further amplified in urban traffic scenarios where vehicle roles and sensing modalities differ substantially. Additionally, as vehicles may frequently join or leave the communication process due to mobility, energy constraints, task prioritization, or unreliable wireless links, this results in biased model updates and slower convergence [17]. This high mobility induces frequent handovers, rapidly time-varying network topologies, and intermittent V2X connectivity, which significantly affect model synchronization and exacerbate communication latency [18].

Nevertheless, the capability of CAV embedded sensors is limited by line-of-sight propagation, i.e., a direct visual path from the vehicle to its surroundings without obstacles. Hence, vehicle-to-everything (V2X) technology is incorporated as a complementary component to embedded sensor systems, enabling the exchange of sensor data between vehicles and their surrounding environment.

**Figure 2:**
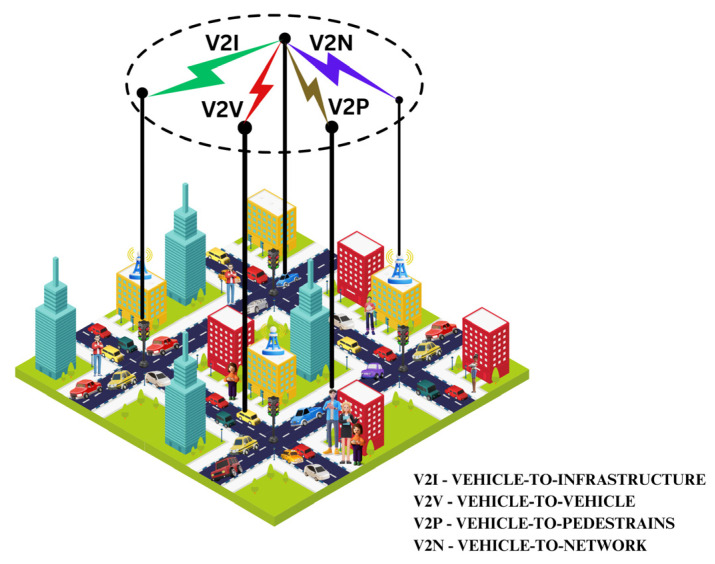
Vehicle-to-everything communication (V2X) architecture.

V2X enhances driver awareness by providing continuous real-time information about on-road threats [19] that they otherwise would be unable to see. The various forms of vehicular communication are illustrated in Figure 2. A vehicle transmits its position, speed, and other parameters to the network and its neighbors; similarly, it listens to what the network and other vehicles are transmitting. This helps considerably in avoiding accidents, road congestion, and any other driving inconvenience. V2X includes mainly two radio access technologies categories: Dedicated Short-Range Communications (DSRC) and Cellular-based vehicle-to-everything technology (C-V2X) [20] (see Table 3).

The IEEE first published the specifications for short-range dedicated communications (DSRC) [19] in 2009 as part of the IEEE 802.11 WLAN set of standards. DSRC technique enables direct inter-vehicular (V2V) and vehicle-to-infrastructure (V2I) communication. Each vehicle transmits securely and anonymously its location, heading, and speed at a frequency of 10 times per second; all neighborhood vehicles receive the message and individually assess the potential risk posed by the transmitting vehicle [21]. While DSRC technology offers key safety applications features in terms of reliability, low latency, robust security, and high-speed communications [22], its limited communication range of approximately 300 m in urban environments and 1000 m on highways [23] narrows its coverage ability.

The cellular vehicle-to-everything (C-V2X) technology is firstly provided with the LTE-Advanced standard (release 14 3GPP) [19]. Compared to DSRC, LTE-V2X communications can leverage higher capacity, broader cell coverage area, and extensively deployed infrastructure to support diverse vehicular communication services. LTE-V2X connects vehicles to each other (V2V), to roadside infrastructure (V2I), to other road users (V2P), and to cloud-based services (V2N) technology [24]. LTE-V2X chipsets are now available for commercial deployment from several manufacturers, including Qualcomm. Nevertheless, the current LTE-V2X on the 4G LTE network does not provide the required low latency for critical V2V communication. Hence, C-V2X technology integrates 5G New Radio technology (Release 15 and Release 16 3GPP). 5G New Radio (5G NR) is an emergent radio access technology designed by 3GPP for the fifth-generation mobile network [25,26]. NR is a physical connection method for radio-based communication, responding to the growing needs for mobile connectivity [25]. NR-V2X is envisioned to incorporate millimeter-wave (mm-wave) spectrum [25] within the 20 to 90 GHz range, especially for applications necessitating short-range communication coupled with high to very high data throughput requirements [27]. Thanks to this feature, NR-V2X offers high-speed communications, up to 10 Gbps, minimal latency reaching as low as 1 ms, and wider coverage [28]. As well, it leverages Mobile edge computing (MEC) to bring services toward the edge of the network, i.e., vehicles and pedestrians. This ensures the acceleration of content, services, and applications by enhancing their responsiveness. Furthermore, the network slicing feature is implemented through deploying virtualization technologies, including network function virtualization (NFV) and software-defined networking (SDN), to operate several logical networks on a common physical network infrastructure [29]. Additional attributes of NR-V2X include diverse transmission mechanisms (e.g., unicast, groupcast, or broadcast), various sidelink operational modes [16], investigation of mechanisms for optimal interface selection (among LTE sidelink, NR sidelink, LTE Uu, and NR Uu) for a given V2X message transmission (Radio Access Technology/Interface selection), and a technical investigation addressing the coexistence of C-V2X and NR-V2X within a single device, i.e., in-device coexistence [25].

**Table 3:** V2X evolution summary.

Refs	Type	Standards	Range of Service				Specification
			V2V	V2I	V2N	V2P	
[21,30]	Basic	DSRC: IEEE 802.11P	x	x			- Low latency to connect vehicles in a distributed manner (direct connection). - Support a limited set of basic safety services.
[19,27]	Extended	LTE/3GPP Rel.14	x	x	x	x	- Increased robustness against interference and enhanced non-line-of-sight functionality. - Improved features (speed, latency, reliability) than DSRC. - Ultra-low-latency, ultra-high reliability and ultra-high bandwidth requirements are not supported.
[25,26]	Advanced	3GPP Rel. 15 and 16	x	x	x	x	- Offer Ultra-low latency, ultra-high bandwidth, ultra-high-reliability V2X services.

## 3. Privacy in Connected and Autonomous Vehicles

### 3.1. Privacy in Literature

**Figure 3:**
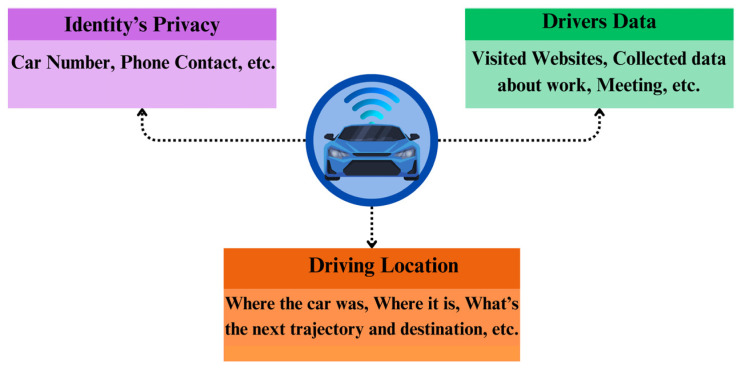
Privacy concerns in CAVs.

In the literature, privacy is defined as a need, a right, a condition, or an aspect of human dignity [31]. It encompasses the right of individuals to control their personal information and the ability to maintain a boundary between someone’s personal data and the external world. An 1890 essay, ‘The Right to Privacy’ [32] by Warren and Brandeis, represents a foundational precursor to modern information privacy law. Within this study, the authors expressed concerns regarding the convergence of nascent instantaneous photography and the extensive circulation of newspapers, which they argued increasingly empowered journalists to intrude upon private matters. They then characterized privacy as the ‘right to be let alone’ and as a fundamental element of ‘the right to one’s personality.’ Within the technological domain, data privacy is defined as a key element of information technology that deals with the capability to identify when, how, and to what extent information is disseminated to others [33]. Within CAV environments, preserving privacy is an indispensable matter (see Figure 3). The spectrum of privacy concerns associated with CAVs includes, but is not limited to, driving location and trajectories, data pertaining to drivers, passengers, and other road users, and usage logs for shared autonomous vehicle services [34,35]. This three-core categorization has been widely adopted in systematization studies on vehicular privacy, although some works further refine these dimensions into behavioral or inference-based privacy risks [35,36].

Only authorized users should be entitled to access and control vehicle-related information, such as a vehicle’s real identity and location profile [37]. Furthermore, authors in [38] claimed that location data can be used in data science to establish associations with identities

Beyond explicit identifiers, time-stamped information can be leveraged to infer sensitive personal attributes, such as residential and occupational locations, age, profession, behavioral patterns, daily routines, and social relationships. Such inferences raise critical privacy concerns, particularly regarding “data bias, data ownership, data use, and data sharing,” which have been recognized as pivotal themes in the politics of CAVs [39].

### 3.2. Introduction of Federated Learning in CAVs

Reliable and real-time traffic flow details are vital for traffic prediction and management in CAVs [40]. However, the dynamic and continuous mobility of CAV networks has always been challenging, i.e., vehicles are characterized by their short connection time, necessitating a dynamic network topology [41]. Thus, investigating real-time data monitoring, prediction of anomalies with high accuracy, and low false rates in CAVs is crucial. Conventional wireless network solutions predominantly rely on parameterized mathematical models and pre-established environmental awareness, which renders them highly vulnerable to the dynamic and unpredictable nature of CAVs, resulting in significant performance degradation.

ML-based solutions [42] can mitigate the uncertainty in the rapidly evolving vehicular network through learning insights from acquired information or by directly engaging with the surroundings to create an accurate policy. The learning process begins by analyzing the given dataset to determine traffic patterns, learn adaptively using mathematical models, and capturing pertinent data to develop optimal predictions [43]. ML has been increasingly deployed in CAV networks to ensure driverless capabilities, road safety prediction, Parking Assistance (PA), and fatigue detection, due to its model-free characteristic, enabling adaptive rapid responses. Nevertheless, conventional ML methodologies require the aggregation of raw vehicular data, which must be uploaded and processed centrally on cloud-based servers or data centers [44]. This centralized paradigm introduces several critical challenges, including transmission overhead, increased latency, heightened risks of information leakage, and privacy exposure risks.

Hence, to address privacy concerns in such dynamic networks, the adoption of distributed privacy-preserving solutions has become mandatory. Federated Learning (FL) has recently emerged as a decentralized ML paradigm [41,45], designed to mitigate transmission overhead while simultaneously enhancing privacy [46]. In this framework, multiple vehicles collaboratively train a shared AI model while retaining their raw data locally, thereby ensuring a notable privacy enhancement [47]. Unlike conventional centralized ML approaches, FL efficiently leverages local computational resources while preserving the privacy of local data [45]. FL associates input values $x_{i}$ to corresponding output labels $y_{i}$ to enable the prediction of unseen data. With an input-output pair ($x_{i}$, $y_{i}$) of size n, the training aim is to determine the model parameters w represented as a vector, by minimizing the loss function $f_{i}(w$), which evaluates the model’s prediction accuracy for the i th data sample using w. Depending on the underlying ML framework, the optimization task can be either convex or nonconvex. As FL is typically implemented with nonconvex neural networks, its finite-sum function optimization algorithm is as follows [48]:

(1) $$\underset{\omega \epsilon R^{d}}{\min}f(w)\;where\;f\left(w\right)≝\;\frac{1}{n}\sum_{i=1}^{n}f_{i}(w)$$

Given that in FL data from distributed nodes remains locally, the global objective function (as presented in Equation (1)) needs to be updated. Assuming K clients participate in the learning iterations, with every participating node carrying $n_{k}$ data samples where $n_{k}$ = |$P_{k}$|. $P_{k}$ represents the data partition allocated to each node K from the entire dataset P. Thus, the new global loss function is expressed as a weighted sum of the local loss functions $f_{k}(w$) as follows [48]:

(2) $$f\left(w\right)=\;\sum_{i=1}^{k}{\frac{n_{k}}{n}F}_{K}\left(w\right)\;where\;f_{k}\left(w\right)=\;\frac{1}{n_{k}}\sum_{i\epsilon p_{k}}f_{i\;}(w)$$

**Figure 4:**
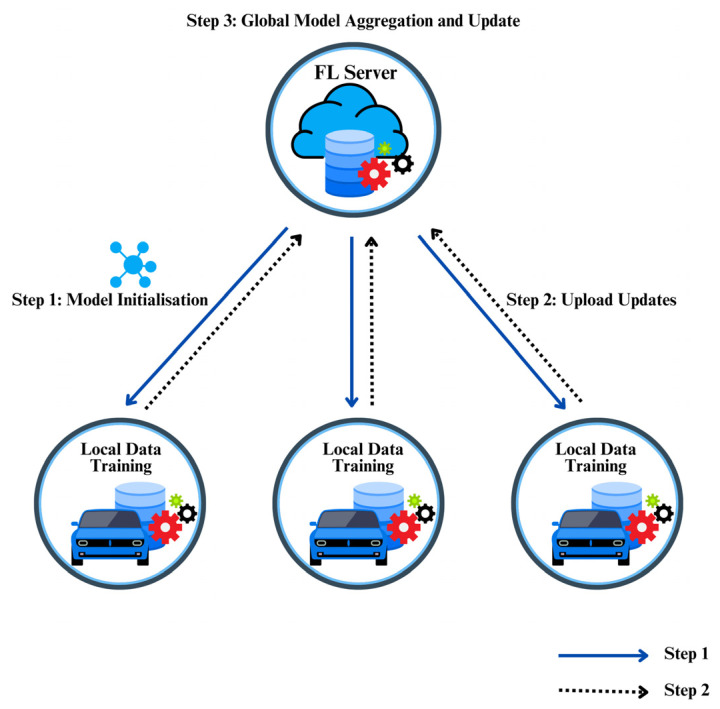
Federated learning for CAVs.

In FL systems, there are two fundamental elements, i.e., the data owners (vehicles) and the FL server acting as the model owner (see Figure 4). Vehicles participate in the training process by using their local models to train an ML model issued by an FL server, and upload the model updates, i.e., the model’s weights, bias, etc., to the server instead of sharing their row data [49]. Local model updates are aggregated by the server to generate a global model [50]. The local model denotes the model trained independently on each participating device, while the global model represents the aggregated model maintained by the FL server. The FL learning procedure proceeds as follows:

Step 1: Task initiation.

The RSU, acting as the server, determines the learning task (the designed activity to perform, and the associated data specifications) and prescribes the hyper-parameters of the global model and the training process. Then, it communicates the initialized global model and task to the designated participating vehicles.

Step 2: Local model training.

Each participating vehicle iteratively trains the received global model by utilizing its local data, thereby updating the local model weights. This procedure is performed iteratively in each round until convergence, i.e., when the optimal parameters that minimize the loss function are retrieved. Subsequently, the resulting local model parameters are transmitted to the aggregator.

Step 3: Global model aggregation and update.

During this step, the RSU gathers all local models transferred from participating vehicles, performs the aggregation process, and subsequently transmits the updated global model parameters back to the vehicles.

The second and third iterations continue until the global loss function attains convergence or an optimal training accuracy is reached. Thus, the global model remains lightweight, as it only operates on local updates, thereby minimizing the number of communicated parameters [46], enhancing communication efficiency, and improving privacy preservation. A conventional and widely adopted algorithm for aggregating local models is the Federated Averaging algorithm, whose pseudocode is presented in Algorithm 1 [48].
**Algorithm 1** Federated Averaging. The K Clients are denoted as k; B, E, and η Represent the Local Minibatch Size, Number of Local Epochs, and Learning Rate, Respectively1: initialize $w_{0}$ 2: **for** each round t = 1, 2, … **do** 3: m ← max (C · K, 1) 4: **Select** a random subset of m clients from the total set of  currently connected and eligible CAVs 5: $S_{t}\;\subseteq \{1,\;2,\ldots ,K\}$ 6: **for** each client k ∈ $S_{t}$ **in parallel do** 7: $W_{t+1}^{k}$ ← ClientUpdate (k, $W_{t}$) 8: $W_{t+1}$ ← $\sum_{k=1}^{K}\frac{nk}{n}\;W_{t+1}^{k}$ 9: **ClientUpdate** (k, w): ‖Run on client k 10: β ← (split $P_{k}$ into batches of size b) 11: β = {$b_{1},\;b_{2}$,…} 12: **for** each local epoch *i* from 1 to E **do** 13: **for** batch b ∈ β **do** 14: w ← w − η▽ℓ (w; b) 15: return w to server

### 3.3. Blockchain

**Figure 5:**
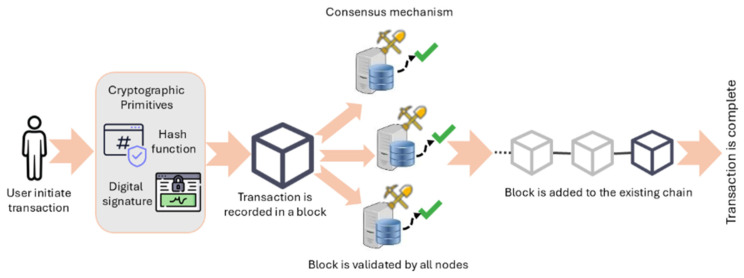
Core architectural components of Blockchain system including cryptographic primitives (e.g., hash functions and digital signatures), decentralized peer-to-peer network architecture, shared digital ledger, consensus algorithms to dynamically validate transactions, validity rules ensuring structural and logical correctness of blocks and transactions, and a container (block) encapsulating transaction data.

Blockchain is defined as a shared and immutable ledger, enabling a secure recording of transactions and the transparent tracking of assets [51]. The term “blockchain” is derived from its method of storing transaction data [11]. It is structured as a list of blocks cryptographically linked via hash values, forming a sequential chain governed by a consensus mechanism, such as Proof-of-Work (PoW). The PoW process is described in Algorithm 2. Consensus process is enabled by miners that validate and append blocks to the chain using digital signatures, thereby ensuring the immutability of the linked blocks against unauthorized modifications and alterations [52]. All network entities respectively monitor any update events and user behaviors in a transparent manner. Through these transaction logs, it is easy for blockchain to identify the origin of model parameter alterations during the training process, which enhances trust among participants [43]. This means that data protection and storage on the blockchain are suitable for sensitive information such as autonomous vehicle communication and user identification. At a conceptual level, blockchain is not built on an entirely new technology, but rather on an integration of existing ones (see Figure 5) [11]: peer-to-peer (P2P) networks, cryptographic algorithms, shared digital ledgers, consensus mechanisms, validity rules, and virtual containers. 

Blockchain is mainly divided into two main categories—permissioned and permissionless [11,52]:

- Permissionless blockchain is characterized by its public and open-access nature; any entity can participate and integrate with the network and participate in the consensus mechanism. In this framework, the identity of participants is masked, which poses a significant security challenge.
- Permissioned blockchain maintains additional constraints on the participating users regarding read access, involvement in the consensus process, or a combination thereof. This controlled access serves to alleviate computational and network communication overhead, which is a primary delay factor.

**Algorithm 2** Proof-of-Work (PoW) consensus**Input**: Candidate block B with transactions **Output**: Valid block with valid nonce 1: **procedure** PROOF_OF_WORK(B) 2:   Nonce ← 0 3:   **while** TRUE do 4:     HashVal ← H(B, Nonce) 5:     **if** HashVal < DifficultyTarget **then** 6:       return (B, Nonce, HashVal)//Valid block found 7:     **else** 8:       Nonce ← Nonce + 1 9:     **end if** 10:   **end while** 11: **end procedure**

#### 3.3.1. Blocks

**Figure 6:**
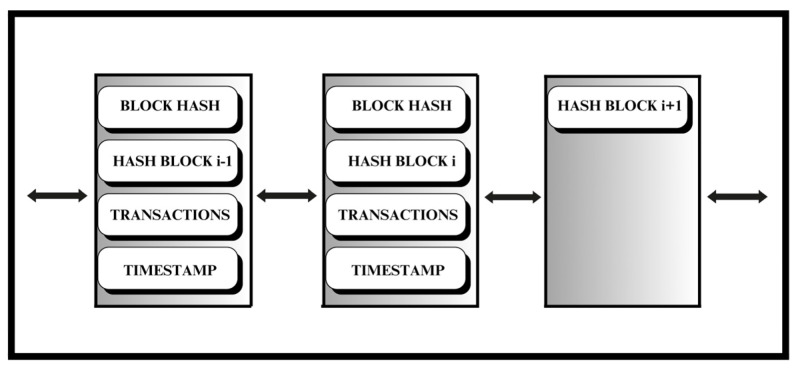
Blocks in a Blockchain.

Blocks record and confirm the transactions based on their time and sequence. Once confirmed, transactions are recorded, guided by a set of pre-agreed rules defined by network participants. Each block within this chain contains a cryptographic hash (a unique digital identifier), time-stamped sets of recent valid transactions, and the hash of the preceding block. The preceding block hash creates a linkage between blocks (see Figure 6), thereby preventing any block alteration or the insertion of a block between existing blocks. Through this mechanism, each successive block reinforces the verification of its predecessor, and hence the integrity of the whole blockchain.

#### 3.3.2. Digital Signatures

Each node is allocated an asymmetric key pair, comprising a private key and a public key [52]. Encryption using the private key necessitates the corresponding public key for decryption, and conversely. The public key acts as the network address allocated to every node, and each digital signatures typically rely on elliptic-curve digital signature algorithms [53].

#### 3.3.3. Consensus Algorithm

Within the peer-to-peer model, nodes are responsible for verifying transactions and incorporating them into the blockchain. This procedure, referred to as mining, constitutes a critical process of the blockchain architecture, as it underpins its decentralized architecture. The core principle of consensus requires that participating nodes perform verification procedures that are computationally intensive to execute but straightforward to verify. This asymmetry serves to deter malicious entities from satisfying the necessary conditions to validate inaccurate transactions.

#### 3.3.4. Hashing

Hashing algorithms are notably fundamental to the blockchain system. As a category of cryptographic algorithms, the hash function is characterized by its ability to process variable-sized input and generate a fixed-length output, known as hashes. SHA family (SHA-1 and SHA-2) are common hashing algorithms [52] within blockchain. A robust hash algorithm must adhere to two critical conditions:

- It should be non-invertible, i.e., computationally infeasible to derive the input from the output.
- The probability of two distinct inputs producing an identical output hash should remain minimal. This condition enhances security, as a minor alteration to the input will change the hash value, thus making tampering easily detectable.

#### 3.3.5. Smart Contract

Firstly proposed by Szabo [54] in 1997, Smart Contracts (SC) are defined as executable computer code that autonomously executes an agreement upon the fulfillment of predefined contractual conditions [43]. These self-executing contracts, stored on a blockchain, provide an automated implementation of an agreement so that every participant can be immediately certain of the outcome [55]. Using SC, blockchain can automate round delineation, ensure trusted dynamic client selections for specified distributed learning services, bypassing a centralized authority, and enable simultaneous aggregation phases for various tasks sourced from distinct device groups [11]. Thus, the complexities and delays of traditional contractual processes, such as integrating the contract within the transaction, are eliminated.

#### 3.3.6. Blockchain Process

**Figure 7:**
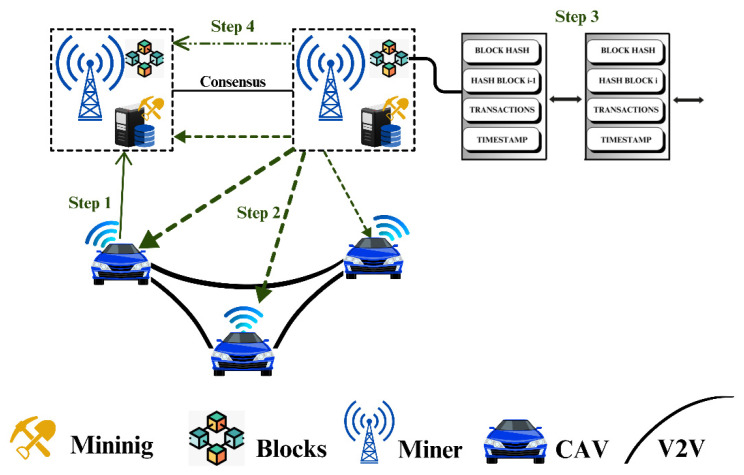
Overview of blockchain operational flow.

A vehicle creates a transaction incorporating the user ID, user signature, and time-stamp as key components (See Figure 7).

- Step 1: The vehicle (task publisher) issues a transaction for a certain request, such as user authentication or cloud service queries, by deploying a SC over the blockchain.
- Step 2: Smart contracts automate transaction authentication, user verification, and asset exchange between service providers and users. SC selects eligible training vehicles to engage in this learning task.
- Step 3: Blockchain miners try to verify the user transaction. Each miner initiates mining until either it determines the hash value or it obtains a generated block from another miner.
- Step 4: If all miners attain an agreement through a consensus mechanism, the validated block and its signature are subsequently added to the blockchain in time-based sequence. Each network node receives this block and synchronizes its blockchain copy accordingly.

### 3.4. Blockchain Integrated with Federated Learning (FLchain)

Federated Learning frameworks have proven effective in addressing key CAV network optimization challenges, such as the communication cost reduction, the efficient allocation of computational and networking resources, and the reinforcement of security and privacy mechanisms [45]. Nevertheless, due to the scalable and dynamic nature of CAV environments, a sole server is incapable of aggregating all updates uploaded from a vast number of vehicles. Consequently, FL faces several challenges during the training time. Since FL requires high communication rounds between the participating vehicles and the server, communication overhead is becoming one of its major bottlenecks [56]. Moreover, relying on a single server to aggregate the parameters issued from the whole network makes FL vulnerable to the single point of failure, which may result in the loss of all network information [11,57]. Thus, it becomes crucial to establish a decentralized FL framework that does not rely on a single central server in order to mitigate the aforementioned issues. Blockchain provides interesting solutions for FL-based CAV networks. Powered by a distributed consensus protocol, blockchain ensures decentralization, immutability, traceability, and impartiality. Blockchain has the ability to avoid the single point of failure issue with a fully decentralized architecture [58]. Blockchain, with the use of customized smart contracts (SC), facilitates the automated delineation, selection, and aggregation phases for multiple FL tasks simultaneously from different distinct node sets.

### 3.5. Privacy Attacks in Federated Learning for CAV Systems

Although federated learning mitigates raw data sharing, it is not yet inherently privacy-preserving and remains vulnerable to several inference attacks. One notable category of threats is gradient inversion and reconstruction attacks, where adversaries can exploit shared model updates to recover sensitive local training data, including images and sensor features [59]. Recent works demonstrate that such attacks remain effective even under practical FL conditions with deep models and partial participation [60]. In the vehicular context, this raises serious concerns, as reconstructed data may reveal private driving scenes, trajectories, or environmental perceptions. Another vulnerability is membership inference attacks, which aim to determine whether a specific data sample was used in a participant’s local training process, thereby leaking sensitive information about vehicle presence, routes, or behavior [59]. Furthermore, property inference attacks enable adversaries to infer high-level attributes of local datasets, such as traffic density patterns or driving styles, without reconstructing individual samples, posing risks to both individual vehicles and fleet operators [61]. Furthermore, recent studies also highlight that CAV-specific characteristics, including non-IID data distributions, sporadic participation, and asynchronous updates, can exacerbate these attacks by introducing update bias and amplifying information leakage through gradients [59]. Collectively, these privacy threats demonstrate that relying solely on federated learning is insufficient to preserve sensitive vehicular data, particularly in large-scale and safety-critical CAV deployments.

### 3.6. Privacy Issues in FL Chain and Proposed Solutions

Malicious vehicles, as well as semi-honest servers, aim to infer local private information related to other network nodes from the offloaded model updates in FLchain. Attackers can exploit private data throughout the training phase or endeavor to re-establish the training set based on the generated gradients (Table 4). To this end, an extensive range of privacy-preserving strategies is investigated to enhance the robustness of FLchain frameworks.

Differential privacy (DP) techniques [62] are applied to locally generated models through the incorporation of artificial noise, thereby perturbing the trained gradients before their offloading to the server. This process reduces the risk of identifying individual records. Subsequently, the server aggregates all clients’ differentially private updates and applies a consensus mechanism to compute the global gradient, which is then broadcast to all participating nodes within each training cycle. Following the global aggregation phase, client-specific updates become irretrievable by any individual node, thereby ensuring privacy preservation during the blockchain-based model exchange [11]. Building upon this concept, the authors in [63,64] present an approach based on two key steps for data handling prior to transmitting trained parameters to the main server. During each training epoch, the aggregator firstly chooses a random set of nodes to refine the global model. Eventually, selected participants will adopt the differential privacy method. In this way, a malicious node will be unable to infer information about other nodes through the use of the shared global model parameters, as it has no indication about the vehicle that has participated in the learning process. Similarly, Authors in [65] introduce a random vehicle—miner association strategy. A malicious node, which in other fixed vehicle–miner association schemes could compromise miners, is unable to exploit this dynamic association mechanism to identify and target a specific vehicle.

Another method proposed in [66] is based on the CPC lightweight encryption algorithm. The CPC algorithm executes eight rounds of encryption, in which the input is a 256-bit plaintext and a 256-bit master key. The plaintext is divided into four subblocks, and 8 subkeys are derived from a master key. In each round of conversion, subblocks will be managed by 4 operations (encryption with a subkey, Count_Zero, loop left shift position, and permutation operation) until generating the ciphertext after the 8 rounds. CPC’s lightweight encryption algorithm provides a secure data interaction among vehicles. Nevertheless, the escalating amount of data leads to a corresponding rise in associated costs, thereby necessitating enhancements in terms of energy consumption and storage capacity.

Furthermore, to mitigate attacks targeting decentralized data sharing mechanisms across multiple entities, the FLchain empowered data sharing scheme is proposed. FL is used to improve computing resource management and increase the effectiveness of the data sharing scheme. However, these methods suffer from high system costs, particularly in terms of latency, while privacy preservation has not been considered [64]. A recent work in [67] proposes integration between Federated learning and Hybrid Blockchain architecture to secure data sharing in Internet of Vehicles (IoV). Federated learning relieves transmission load and addresses the privacy concerns of data providers. The Hybrid Blockchain architecture (PermiDAG) comprises the integration between a permissioned blockchain and a local Directed Acyclic Graph (DAG) to improve the security and reliability of model parameters. In PermiDAG, the permissioned blockchain operates within RSUs, while the local DAG operates on the vehicles. The core permissioned blockchain records all inter-vehicle data-sharing transactions, including details about consumers, providers, data profiles, model parameters in the global aggregation, and a summary of transactions in the local DAG. However, this technique introduces communication overhead and potential bottlenecks under high transaction volumes.

Other solutions based on homomorphic encryption [68] are relevant for outsourced storage privacy-preserving and computation in the FL training process, where data can be encrypted prior to being shared on the blockchain. This is a crucial technique for FLchain applications, notably within the healthcare domain [69], in which the highly sensitive nature of health data and personal information necessitates rigorous privacy protection.

**Table 4:** Privacy issues in FLchain and current solutions.

Privacy Attacks in FLchain	Solution	Limitations
Exploit information from the shared model (data privacy during whole decentralized learning)	Differential privacy techniques applied to locally generated models with artificial noise (Perturbing data) [62].	- Effectiveness is limited by the inherent privacy–utility trade-off. - The absence of adversarial attack simulations limits the empirical validation of the claimed privacy guarantees.
	Differentially private and selective participants; Privacy-protection solution for participants by two main steps: 1. server first chooses a random number of vehicles to train the global model. 2. Perturbe the trained model by incorporating noise prior transmitting the parameters to the server [10,63].	- Random vehicle selection may systematically exclude data from rare driving scenarios, reducing model generalizability in safety-critical CAV applications. - Attack simulations have not been provided.
Attacks on user’s privacy while sharing information	CPC lightweight encryption algorithm [66].	High energy consumption and storage cost. Frequent encryption and decryption operations amplify battery drain and increase latency.
Attacks on data sharing mechanism for distributed multiple parties	Blockchain empowered data sharing framework improves security during the sharing process without requiring a centralized trust authority. FL improves the utilization of computing resource and increase the efficiency of the data sharing scheme [64,67].	High system costs including end-to-end training latency, e.g., consensus delays, block confirmation time, and smart contract execution.
Attacks on outsourced storage and computation in FL training	Homomorphic encryption [68] where data can be encrypted before sharing on the blockchain for FL model aggregation.	High computational complexity and communication overhead, i.e., require multi-round interactions and increase aggregation latency.

## 4. Permissioned Blockchain Integrated with Federated Learning for Privacy in CAVs (Permi-FLchain)

Blockchain has emerged as a potential technology that provides distributed secure solutions in FL by offering tamper-proof, anonymity, and traceability capabilities. Current solutions based on the implementation of FL with blockchain are effective in enabling secure data sharing in CAVs, as discussed in Section 3.4. The implementation of smart contracts enables a secure exchange of global ML models between RSUs and distributed training vehicles, thereby enabling consensus on update verification and ensuring transparency throughout the FL updating process. This mechanism enforces unbiased and tamper-resistant data handling, thus enhancing the safety and reliability of the FL framework against external data threats [11].

Nevertheless, how to adeptly guarantee privacy in FL by using the blockchain technique remains an open issue that needs to be further discussed [70]. To restrict access to the network and mitigate the loss of control over private information, blockchains can be categorized into three main types: permissioned, permissionless, or consortium blockchain [11].

1. The consortium blockchain is configured by a partnership of two or more than two organizations and can include both permissioned and permissionless networks [71].
2. Permissionless blockchain is open to any entity to join the chain without a particular identity, and it does not require permission to participate in the training process. However, while permissionless blockchain provides a fully decentralized and authority-free operation, it remains hindered by insufficient privacy and anonymity assurances [71].
3. Permissioned blockchain deploys an access control layer that restricts specific actions to only authorized and identifiable vehicles. It is composed of three types of nodes: participating vehicles, a certification authority that provides the digital certificate, and auditors.

Within a permissioned blockchain, vehicles can log with the certificate authority, either an RSU or an MBS, to be authorized as trusted vehicle nodes of the permissioned blockchain. Furthermore, to participate in the training process, vehicles require a certificate to join the V2I data sharing. A digital certificate enables the reliable identification of information, ensures resistance to forgery, and guarantees authenticity due to its provenance from a recognized issuing authority. Every participant owns a unique identity, which ensures the use of policies to restrain network participation and access to transaction details. Through the use of IDs and permissions provided by a trusted authority (also called super nodes), vehicles can specify which transaction details they want other participating vehicles to be allowed to view. Permissions can be assigned to specific users, such as auditors who require access to further transaction details. Upon an auditor or regulator joining the network, cryptographic confidentiality services, enabled by digital certificates, provide these entities with exclusive access to the full set of transaction records.

Hence, permissioned blockchain restricts participation to authenticated CAV entities, enabling low-latency consensus, fine-grained access control, and regulatory accountability. Compared to permissionless blockchain, permissioned designs significantly reduce consensus overhead and privacy exposure, making them more compatible with safety-critical vehicular applications [72]. Operationally, participating entities in Permi-FLchain perform local model training on their onboard data and periodically submit model updates to an FL coordinator, typically deployed at RSUs or edge servers. A permissioned blockchain layer governs participant authentication, access control, and update logging, where only authorized entities, such as infrastructure providers or trusted mobility operators, can act as validators. Model updates are recorded on-chain via smart contracts, enabling verifiable aggregation, traceability, and resistance to tampering without exposing raw data [72]. Hence, Permi-FLchain ensures data privacy by keeping sensitive vehicular data on-device, while the permissioned ledger limits adversarial participation through the use of permissions. However, practical deployment remains challenging. Continuous transaction logging leads to ledger storage growth, which exceeds the capacity of resource-constrained vehicles and necessitates off-chain storage or pruning mechanisms. Although consensus delay is reduced, it can still impact time-sensitive applications under high transaction rates. Moreover, certificate authority placement is crucial as RSU or edge-based validators improve latency but increase vulnerability to localized failures, while wider distribution enhances robustness at the cost of higher communication overhead. These challenges motivate adaptive consensus protocols and edge-aware blockchain designs tailored to vehicular environments [73].

Most Permi-FLchain frameworks for CAVs adopt a multi-adversary threat model that captures the semi-trusted and highly mobile nature of vehicular networks. Vehicles are modeled as either honest-but-curious participants that may attempt inference on received model updates, or as fully malicious agents capable of model-poisoning, free-riding, or data-exfiltration through crafted local gradients [72]. RSUs and edge servers orchestrating FL are often assumed to be honest-but-curious, faithfully executing the protocol while opportunistically extracting sensitive information from aggregated parameters [49]. External adversaries can eavesdrop, replay, or launch denial-of-service attacks, yet are not capable of breaking standard cryptographic primitives [72]. Under this threat model, Permi-FLchain aims to protect raw vehicular data confidentiality and ensure the integrity and traceability of model updates.

## 5. Software-Defined Vehicular Networking in CAVs

**Figure 8:**
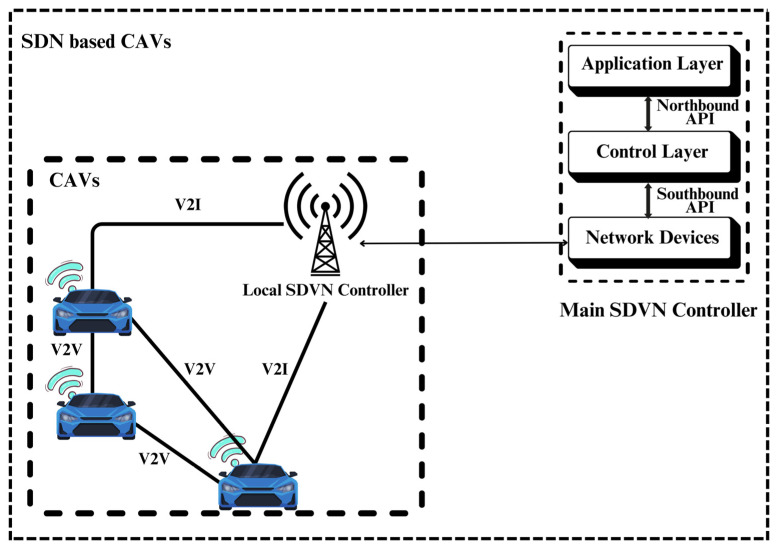
SDN-based CAVs architecture.

Deploying Software-Defined Networking (SDN) within CAVs has attracted growing attention within the research community [74,75]. SDVN architecture incorporates SDN and CAVs to create a safe and robust driving environment [76] (see Figure 8). SDVN-enabled wireless networks are emerging as programmable and highly adaptable platforms for privacy-preserving solutions. By decoupling the data and control planes, SDVN enables centralized orchestration of privacy-preserving mechanisms. It introduces a logically centralized and virtualized controller characterized by two principal attributes: programmability, which allows dynamic configuration and management of network resources, and flexibility, which enables RSUs and BSs to mainly focus on connectivity and flow-table operations while the controller orchestrates higher-level network functions [77]. To enable the controller communication with the rest of the network, northbound and southbound APIs are employed. The southbound API enables communication between the SDVN controller and the underlying vehicular network infrastructure, allowing the controller to configure devices, install forwarding rules, and collect network state information. In contrast, the northbound API provides an interface between the controller and network applications, providing high-level abstractions through which applications can express policies, requirements, or service intents.

In summary, SDVN enhances the selection of optimal routes, optimizes network programmability for critical vehicular applications [78], and introduces privacy and security measures [79]. While some studies have focused on resolving security vulnerabilities within the control plane [80], including malware attacks and distributed denial-of-service, the privacy challenges have not received too much focus.

### 5.1. Data Routing in SDVN

To obtain deeper insights into privacy-preserving measures within SDVN, it is essential to understand the underlying routing process, as it directly influences communication efficiency, security, and network management. Routing within SDVN, as described in Algorithm 3, operates according to the following detailed aspects. Firstly, the vehicle initiating a task requires transmitting association requests to determine the optimal RSU to connect with. In SDVN, it is possible to get several candidate RSUs within the vehicle’s communication range. The best RSU is typically the one with minimal traffic load and a robust communication link [81]. To measure the quality of RSU connections, several methods, such as Kriging weights [82], are used. When the RSU is selected, the vehicle stores its requests in a queue. Each request is transmitted sequentially to the SDVN controller [83]. The RSU serves as a local controller in order to preserve the network structure informed by periodic vehicular beacons or trajectory/link prediction models, including the velocity, location, direction, and so on. The entire network can be segmented into distinct zones, where in each individual zone, local controllers exchange and update regional network topology data with the main controller.

Through logically centralized network visibility, SDVN controllers can dynamically configure V2X forwarding paths, prioritize latency-sensitive traffic, and coordinate communication via roadside units (RSUs) and edge servers, thereby improving network efficiency under high mobility [84]. When a source vehicle sends a route query, the controller, leveraging knowledge of the network topology, assumes full responsibility for determining the path from source to destination. Routes computation is based on the weights of links based on routing metrics [80]. Some shortest path algorithms are applied for this purpose, such as Dijkstra [83], Bellman-Ford, and Floyd algorithm [85]. To ensure the delivery of a data message, the controller can generate several path options for each source-destination communication. Routing paths are subsequently sent back to the SDVN controller. Generally, services in the global controller/Cloud are classified into safety and non-safety messages. A scheduling algorithm prioritizes emergency messages considering their deadlines and sizes. The message with the shortest deadline and minimal length will be assigned a higher priority among all services. Hence, the messages are transmitted back to the SDVN controller, ordered by their assigned priorities. For non-safety messages, the requests are classified using the First-Come First-Serve (FCFS) scheduling algorithm [83].
**Algorithm 3** SDVN-Based Routing Process for CAVs**Input:** Vehicle task request Req, Nodes u, v **Output:** Optimal routing path 1: **procedure** Vehicle_Association (Req) 2:   CandidateRSUs ← ScanCommunicationRange (Vehicle) 3:   **for** each RSU in CandidateRSUs **do** 4:     LinkQuality (RSU) ← EvaluateLink (RSU) 5:     TrafficLoad (RSU) ← GetTrafficLoad (RSU) 6:   **end for** 7:   BestRSU ← argmin_{RSU}(TrafficLoad (RSU), LinkQuality (RSU)) 8:   QueueVehicleRequest (BestRSU, Req) 9:   Transmit (Req, SDVN_Controller) 10: **end procedure** 11: **procedure** Routing (Req, TopologyData) 12:   Source ← ${Req}_{source}$ 13:   Destination ← ${Req}_{destination}$ 14:   **for** each link (u, v) in TopologyData **do** 15:     Weight (u, v) ← ComputeWeight (u, v) 16:   **end for** 17:   Paths ← ShortestPathAlgorithms (Source, Destination, Weight) 18:   Send (Paths, SDVN_Controller) 19: **end procedure**

### 5.2. Privacy in SDVN

SDVN has a direct impact on privacy-aware communication and traceability in CAVs by enabling fine-grained enforcement of privacy policies at the network level, such as adaptive access control, traffic isolation, pseudonym management, and selective forwarding of sensitive data flows. For instance, authors in [86] proposed a weighted conditional model based on two stages for an anonymous authentication approach. Leveraging the global planning and configuration capabilities of SDN, vehicles are categorized in distinct priority levels based on weighted values, resulting in limiting malicious vehicle participation. Upon a vehicle’s entry into a local controller’s management range and request for message signing and forwarding, the controller will select the most suitable candidate forwarding sets (CFS) and transmit global parameters to it. The signed message, along with the attached CFS, is then transmitted by the vehicle. Neighboring vehicles verify their inclusion within the CFS. If in, they validate the message and determine whether to discard or forward it.

Another privacy-aware authentication strategy by exploring the infrastructure of 5G software-defined vehicular network is introduced in [87]. Authors use elliptic-curve public-key cryptography with two levels of pseudonyms, PPID and SPID, and a registration list. Before joining the network, vehicles must register with the Department of Motor Vehicles (DMV) by submitting official documentation that verifies their true identity. Consequently, a token, rather than the user’s real identity, is employed between the Trusted Authority (TA) and the DMV to preserve the user’s personal data. Following the registration procedure, each vehicle is issued a PPID and a public-key certificate. Then, a vehicle transmits a request to get SPIDs using its PPID. The PPID is adopted by participating vehicles to ensure communication with the TA. The SPID is used for V2V communications. In [88], the authors aim to guarantee location privacy using a self-privacy- preserving architecture that leverages location privacy management in vehicular networks through software-defined techniques. The control plan dynamically selects the Pseudonym-Changing Strategies (PCSs) based on the mobility, topology, and attacker models. The data layer then converts these established rules into actions to implement the PCSs.

Authors in [89] proposed an access control model and authentication for SDVN controllers, using an interconnected blockchain sub-network architecture. An access management and inter-subnetwork authentication mechanism is designed for all SDVN entities, including vehicles, roadside equipment, and controllers. Although authentication serves as a fundamental defense mechanism, ensuring that an incoming message is only generated from an authenticated node is challenging. Certain compromised vehicles, even after successful authentication, may pose as legitimate CAV users and transmit falsified messages with the intent of disrupting network communication. Consequently, authors in [90] propose a collaborative intrusion detection system (IDS), besides an authentication schema. First, within an RSU-driven group authentication framework, every vehicle in the RSU coverage area is provided with a group ID-key pair from the RSU to enable further safe communication among vehicles. Secondly, to detect potential intrusions within the network, SDVN uses a collaborative learning approach that preserves privacy by leveraging an integration of homomorphic encryption and differential privacy schemes, as summarized in Table 5.

However, these capabilities come at the cost of introducing new privacy and security risks associated with controller centralization. The SDVN controller represents a high-value attack target, as it may aggregate fine-grained information about vehicle identities, locations, communication patterns, and learning participation, creating a single point of privacy leakage if compromised [84]. Even under an honest-but-curious threat model, centralized controllers can infer long-term vehicle trajectories, behavioral profiles, and sensitive correlations between network flows and learning activities. Furthermore, a controller can enable large-scale surveillance, selective censorship, or privacy policy manipulation across the entire vehicular network [84]. These risks are exacerbated in large-scale CAV deployments, where controller overload, delayed policy enforcement, or synchronization failures can further undermine privacy guarantees. As a result, recent research increasingly emphasizes the need for distributed or hierarchical SDVN control, controller accountability mechanisms, and privacy-aware controller designs that limit data exposure while preserving the benefits of network programmability [84].

## 6. Toward Optimal Permi-FLchain System for CAV

As previously discussed, several techniques have been proposed to ensure an efficient privacy-preserving model within CAV environments. FLchain architecture proves its effectiveness within CAVs in terms of transparency, immutability, and privacy-preserving. However, FLchain inherently faces the delay challenge due to the long blockchain system management, including end-to-end delay analysis, consensus, and computation delays. In other words, delay $T_{tot}$ is calculated through aggregating the transmission delay of the entire system and the mining consensus delay, comprising block generation and verification. In order to minimize the overall delay $T_{tot}$, the communication delay, i.e., uploading the local model and downloading the global model, the blockchain delay, i.e., block arrival, propagation, and verification delays, and forking probability should be minimized. The total latency $T_{\mathrm{tot}}$, can be expressed as the sum of communication $T_{comm}$ and blockchain consensus delays $T_{bc}$

(3) $$T_{tot}=T_{comm}+T_{bc}$$

where $T_{comm}$ consists of the uplink delay for transmitting local model updates from CAVs to the aggregator and the downlink delay for disseminating the updated global model:

(4) $$T_{comm}=\;\sum_{k\in S}\left(\frac{w_{k}}{R_{k}^{up}}+\;\frac{w}{R_{k}^{down}}\right)$$

Here $w_{k}$ denotes the size of the local model update from client k, w is the global model size, and $R_{k}^{\mathrm{up}}$ and $R_{k}^{\mathrm{down}}$ represent the uplink and downlink data rates, respectively.

The blockchain delay $T_{\mathrm{bc}}$ is composed of block generation, propagation, and verification delays:

(5) $$T_{\mathrm{bc}}=T_{\mathrm{gen}}+T_{\mathrm{prop}}+T_{\mathrm{ver}}$$

where $T_{\mathrm{gen}}$ is the consensus delay, $T_{\mathrm{prop}}$ denotes block propagation delay and $T_{\mathrm{ver}}$ represents block validation and signature verification time. Forking probability further impacts $T_{\mathrm{bc}}$ by introducing retransmissions or consensus retries.

- Communication delay reduction

Leveraging 5G New Radio (5G-NR) within CAVs can enhance performance regarding reliability, throughput, capacity, latency, and mobility to support the V2X communications. Services supported by 5G-NR are categorized into three primary types: Ultra-Reliable Low-Latency Communications (URLLC), massive Machine-Type Communications (mMTC), and enhanced Mobile Broad Band (eMBB) [91].

Ultra-reliable low-latency communications (URLLC) provide instantaneous and optimized systems for processing high-volume data with minimal delay. MIMO and MEC are two major technologies in the 5G system [92,93], used to ensure URLLC for V2X communication. MEC strategically relocates computing power closer to end-users to support applications and services requiring unique connectivity features such as ultra-low-latency and ultra-high reliability.

Massive Machine-Type Communications (mMTC) is designed to enable massive connectivity between distributed nodes [77]. Massive MIMO and Network slicing are key enablers for mMTC.

Enhanced Mobile Broadband (eMBB) is an indispensable service category of 5G that establishes a minimum data transfer rate, promising to deliver both increased bandwidth and reduced latency. eMBB is intended to support data-intensive applications such as 4 K video streaming, virtual reality, and augmented reality [93]. Technically, eMBB is provided through mmWave range to achieve high bandwidth allocations, massive MIMO composed of several TX/RX antennas to enable high user throughput and high spectral efficiency, and spectrum sharing to unlock more spectrum and extend the 5G network.

- Blockchain delay reduction

**Figure 9:**
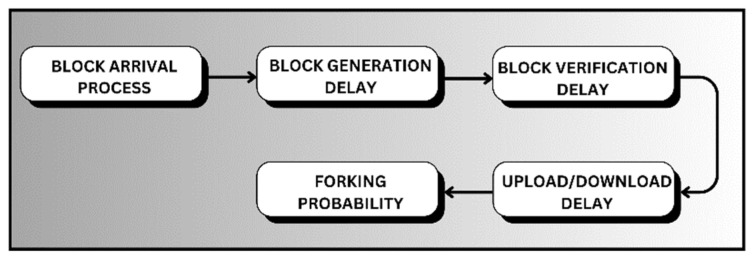
Total blockchained system delay.

The total system delay’s expected value is defined as the combination of the mining consensus and the communication within the entire system. Considering relevant insights into system delay, the delay of the consensus algorithm [65] should be kept minimal in order to reduce the overall blockchain delay. The consensus process directly impacts the block generation and propagation delay and forking probability (See Figure 9). Classical consensus protocol, e.g., PoW, brings high computation cost, communication overhead [34], and provides a minimal additional contribution to FL. Consequently, it reduces the practical applicability of the FL-based CAV system.

To tackle this challenge, a novel consensus is proposed in [62], which is based on the accuracy performance of each consensus node in the testing dataset. During each round, every consensus node i∈{1,2,…,N} gathers the local updates and generate a preliminary aggregation model $M_{i}$. The testing dataset $D_{test}$ is first predicted using the self-assessed reliability of each node’s model within the collaborative training framework. The reliability of each node is then quantified by the prediction accuracy Acc, denoted as

(6) $${Acc}_{M_{i}}=\frac{1}{D_{test}}\sum_{x\in D_{test}}1(fM_{i}\left(x\right))$$

where $fM_{i}(x)$ represents the predicted label of input x using model $M_{i}$, and 1(⋅) is the indicator function. The consensus node $i^{*}$ with the highest testing dataset consensus accuracy will be designated as the leader.

(7) $$i^{*}={max}_{i\in \left\{1,\ldots ,N\right\}}Acc(M_{i})$$

The difference between accuracy consensus and traditional consensus like PoW, is that once the accuracy of preliminary aggregation is calculated, the leader generates a block with the highest accuracy performance. This block will consequently be processed by other consensus nodes for approval. However, in PoW, all miners should mine the block, and therefore, the consensus process takes a longer delay and brings a high computation cost.

While the accuracy consensus design improves energy efficiency and learning relevance, it introduces a range of substantial security vulnerabilities. Notably, accuracy manipulation allows malicious or selfish participants to craft poisoned local updates that artificially inflate validation accuracy on shared or biased evaluation datasets, thereby increasing their probability of being elected as the leader [49]. Such manipulation is particularly feasible in non-IID federated settings, where heterogeneous data distributions can distort global accuracy measurements. Additionally, collusion attacks pose a significant threat, where a subset of nodes may coordinate to submit mutually reinforcing updates or selectively validate each other’s models, effectively biasing the consensus process and undermining its fairness and robustness [18]. These risks demonstrate that accuracy-based consensus mechanisms, while promising, require complementary security considerations such as robust aggregation, multi-metric validation, reputation systems, or cryptographic verification to ensure secure and trustworthy operation in adversarial federated environments.

- Forking probability

Forking issue is produced if one miner receives simultaneously two or more satisfied models from distinct miners. Authors in [58] propose to use the highest reputation rule to eliminate any forking probability. This rule consists of selecting the miner possessing the highest reputation within the network. Thus, it guarantees that all miners start mining at the same time and eliminates any forking probability. Furthermore, SDVN can improve this process by providing enhanced network programmability, centralized control, and virtualization of functions, which collectively improve consensus coordination and further mitigate the risk of forks.

**Figure 10:**
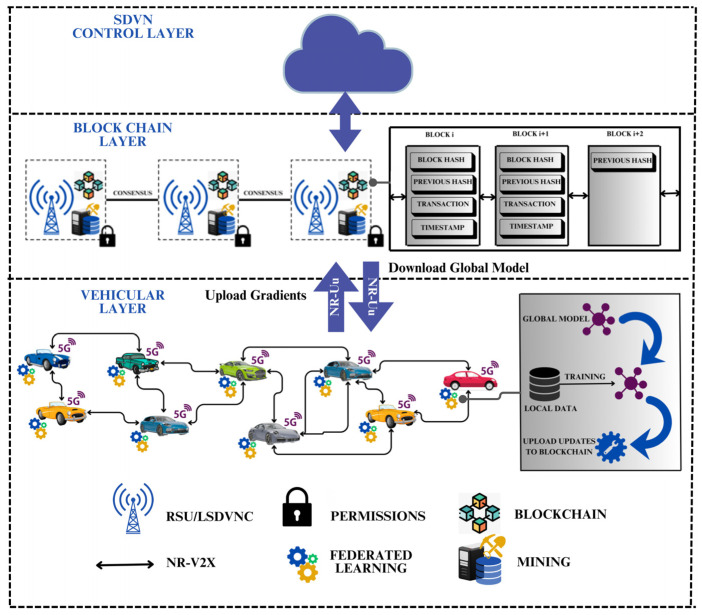
Permissioned blockchain integrated with federated learning in SDVN.

Based on the previous discussion, a proposed privacy-preserving architecture is illustrated in Figure 10. The architecture integrates Federated Learning to ensure raw data remains local, thereby reducing privacy risks, and incorporates 5G-NR to support high-reliability, low-latency communication. Blockchain is employed to establish a decentralized framework that mitigates the risk of a single point of failure, while SDVN contributes additional capabilities in terms of network programmability, scalability, and flexible management of CAV functionalities.

## 7. Future Research Directions

Despite significant advances in privacy-preserving mechanisms for connected and autonomous vehicles (CAVs), several critical research gaps and open challenges remain unresolved. A key challenge lies in balancing system scalability and privacy robustness. Although permissioned blockchain substantially reduces consensus overhead compared to permissionless blockchain, they still incur significant latency, storage expansion, and coordination costs that conflict with the stringent real-time requirements of safety-critical CAV applications [94]. Similarly, integrating federated learning (FL) with robust cryptographic primitives, such as homomorphic encryption or secure multi-party computation, remains computationally prohibitive, particularly in large-scale and highly mobile vehicular networks with non-IID data distributions [36]. Another challenge concerns trust management in semi-decentralized architectures, where entities such as roadside units (RSUs), SDVN controllers, and consortium blockchain validators are often assumed to be trustworthy, despite their susceptibility to insider threats and coordinated attacks [36]. Moreover, long-term and cumulative privacy leakage caused by repeated model updates, trajectory correlation, and imperfect pseudonym-changing strategies remains insufficiently considered, particularly when learning, communication, and control layers interact over extended operational periods [36]. Future research should therefore prioritize the development of integrated privacy-by-design architectures that jointly optimize FL, blockchain, and SDVN components, rather than treating them as loosely coupled modules. In particular, adaptive and context-aware privacy mechanisms capable of dynamically tuning privacy budgets, client participation strategies, and consensus parameters in response to network conditions, mobility patterns, and threat levels, represent a promising research direction [95]. Another critical direction to consider is the design of robust and verifiable FL aggregation schemes that are resilient to poisoning, inference, and collusion attacks under permissioned blockchain governance, while maintaining acceptable latency and model accuracy [60]. Additionally, incorporating lightweight trust and reputation management mechanisms into SDVN control planes may help mitigate insider threats and reduce reliance on computationally expensive cryptographic solutions. Finally, there is a pressing need for large-scale, realistic testbeds and benchmarking frameworks that evaluate privacy-preserving CAV solutions under heterogeneous mobility, adversarial behaviors, and evolving regulatory constraints, thereby bridging the gap between theoretical privacy guarantees and deployable real-world systems [60].

## 8. Conclusions

The evolution of the transportation sector, supported by the integration of connected and autonomous driving technologies, represents a transformative journey aimed at enhancing overall drivers’ experiences by improving both efficiency and safety. In the context of this evolution, this paper has firstly presented a critical analysis of Connected and Autonomous Vehicles infrastructure, most commonly occurring privacy threats, in addition to a literature review on the privacy-preserving methods. To tackle this, innovative solutions such as Differential Privacy (DP), Federated Learning (FL), Homomorphic encryption, etc., have been explored. Additionally, the integration of blockchain has shown promise not only in providing a decentralized architecture but also in ensuring users’ privacy. Indeed, it is crucial to recognize that enhancing existing privacy-preserving solutions requires addressing the continuously evolving threats and challenges associated with the dynamic nature of CAVs. The journey toward secure and intelligent CAVs demands ongoing research, collaboration, and adaptation to guarantee that privacy remains a foundational aspect.

## Figures and Tables

**Table 1:** Summary of acronyms.

Notation	Meaning
ADAS	Advanced Driver Assistance System
CAVs	Connected and Autonomous Vehicles
FL	Federated Learning
BC	Blockchain
BS	Base Station
AP	Access Point
SDVN	Software-Defined Vehicular Networking
IoV	Internet of Vehicles
ECU	Engine Control Unit
MAC	Medium Access Control
RSU	RoadSide Unit
3GPP	Third Generation Partnership Project
RAT	Radio Access Technology
V2P	Vehicle to Pedestrian
V2I	Vehicle-to-Infrastructure
V2N	Vehicle to Network
M	Meters
LTE	Long-Term Evolution
NR	New Radio
PoW	Proof-Of-Work
SC	Smart Contracts
FCFS	First-Come, First-Served
C-RAN	Cloud-Radio Access Network
WHO	World Health Organization
FLchain	FL Integrated with BC

**Table 2:** ADAS system.

Sensor	Range	Purpose
Long-range RADAR	~250 m	Adaptive Cruise Control
LiDAR	~150 m	Collision avoidance, Pedestrian detection, Emergency brake
Camera	~80 m	Surrounding view
Short/medium range RADAR	~20 m	Blind spot Detection, Cross traffic alert, Oncoming traffic
Ultrasonic	2–4 m	Park Assist

**Table 5:** Privacy issues and proposed solutions in SDVN.

Privacy Issue	Solution	Limitations
Communication hacks	Weight-based conditional anonymous authentication scheme [86].Message issued from a ${vehicle}_{i}$ have to be verified based on the vehicle’s classification.	Semi-honest vehicles can provide misleading information, selectively drop the network packets, or exploit their authenticated status to conduct insider attacks.
User’s privacy attacks	Privacy-preserving authentication scheme [87]. Elliptic-curve public-key cryptography and a registration list.	The validation time of SPID is not taken into consideration to calculate the message delay, which can significantly increase end-to-end message latency in high-density traffic scenarios.
	Software-Defined Location Privacy Protection for Vehicular Networks [88]. The control plan dynamically selects the Pseudonym-Changing Strategies (PCSs).	PCS relies heavily on a centralized or logically centralized control plane, which may become a performance bottleneck or a high-value attack target.
Access control attacks	A Scalable Blockchain-based Approach for SDVN devices Authentication and Access Control [89].	Non-negligible consensus and transaction confirmation delays are problematic for real-time vehicular access control.
Inference attacks	Privacy preservation mechanism applied to the learning model using a combination of differential privacy (DP) and a homomorphic cryptosystem. Additionally, the RSU-based group authentication technique is used to prevent outside attackers [90].	The collaborative learning model increases computational complexity, communication overhead, and aggregation latency.

## Data Availability

The original contributions presented in this study are included in the article. Further inquiries can be directed to the corresponding author.

## References

[1] Cao Y., Ahmad N., Kaiwartya O., Puturs G., Khalid M. (2018). Intelligent transportation systems enabled ICT framework for electric vehicle charging in smart city. Handbook of Smart Cities: Software Services and Cyber Infrastructure.

[2] Hassan A.N., Kaiwartya O., Abdullah A.H., Sheet D.K., Raw R.S. (2018). Inter vehicle distance based connectivity aware routing in vehicular adhoc networks. Wirel. Pers. Commun..

[3] US Department of Transportation (2017). Vehicle-to-Vehicle Communication Technology. https://www.nhtsa.gov/sites/nhtsa.dot.gov/files/documents/v2v_fact_sheet_101414_v2a.pdf.

[4] Rana M.M., Hossain K. (2023). Connected and autonomous vehicles and infrastructures: A literature review. Int. J. Pavement Res. Technol..

[5] Kaiwartya O., Abdullah A.H., Cao Y., Altameem A., Prasad M., Lin C.T., Liu X. (2016). Internet of vehicles: Motivation, layered architecture, network model, challenges, and future aspects. IEEE Access.

[6] Checkoway S., McCoy D., Kantor B., Anderson D., Shacham H., Savage S., Koscher K., Czeskis A., Roesner F., Kohno T. (2011). Comprehensive experimental analyses of automotive attack surfaces. Proceedings of the 20th USENIX Security Symposium, San Francisco, CA, USA, 8–12 August 2011.

[7] Mwanje M.D., Kaiwartya O., Jha D.N., Khasawneh A.M., Sadiq A., Cao Y. (2025). Misbehaviour Prediction in CAV Network using Aggregated Trust Analysis. Int. J. Intell. Transp. Syst. Res..

[8] Sharma P., Austin D., Liu H. (2019). Attacks on machine learning: Adversarial examples in connected and autonomous vehicles. Proceedings of the IEEE International Symposium on Technologies for Homeland Security, Woburn, MA, USA, 5–6 November 2019.

[9] Tan K., Bremner D., Le Kernec J.L., Imran M. (2020). Federated machine learning in vehicular networks: A summary of recent applications. Proceedings of the 2020 International Conference on UK–China Emerging Technologies (UCET 2020), Glasgow, UK, 20–21 August 2020.

[10] Lim W.Y.B., Luong N.C., Hoang D.T., Jiao Y., Liang Y.-C., Yang Q., Niyato D., Miao C. (2020). Federated learning in mobile edge networks: A comprehensive survey. IEEE Commun. Surv. Tutor..

[11] Nguyen D.C., Ding M., Pham Q.-V., Pathirana P.N., Le L.B., Seneviratne A., Li J., Niyato D., Poor H.V. (2021). Federated learning meets blockchain in edge computing: Opportunities and challenges. IEEE Internet Things J..

[12] Fu Y., Yu F.R., Li C., Luan T.H., Zhang Y. (2020). Vehicular blockchain-based collective learning for connected and autonomous vehicles. IEEE Wirel. Commun..

[13] Silva C., Masini B., Ferrari G., Thibault I. (2017). A survey on infrastructure-based vehicular networks. Mob. Inf. Syst..

[14] Chen S., Hu J., Shi Y., Peng Y., Fang J., Zhao R., Zhao L. (2017). Vehicle-to-everything (V2X) services supported by LTE-based systems and 5G. IEEE Commun. Stand. Mag..

[15] Zhu L., Yu F.R., Wang Y., Ning B., Tang T. (2018). Big data analytics in intelligent transportation systems: A survey. IEEE Trans. Intell. Transp. Syst..

[16] Ye H., Liang L., Li G.Y., Kim J., Lu L., Wu M. (2017). Machine Learning for Vehicular Networks. arXiv.

[17] Khalil H.K. (2002). Nonlinear Systems.

[18] Mekki T., Jabri I., Rachedi A., Chaari L. (2022). Software-Defined Networking in Vehicular Networks: A Survey. Trans. Emerg. Telecommun. Technol..

[19] Gyawali S., Xu S., Qian Y., Hu R.Q. (2021). Challenges and solutions for cellular-based V2X communications. IEEE Commun. Surv. Tutor..

[20] Dibaei M., Zheng X., Xia Y., Xu X., Jolfaei A., Kashif Bashir A., Tariq U., Yu D., Vasilakos A.V. (2021). Investigating the prospect of leveraging blockchain and machine learning to secure vehicular networks: A survey. IEEE Trans. Intell. Transp. Syst..

[21] Zbairi M., Ez-Zazi I., Arioua M. (2019). Ultra-reliable 5G millimeter-wave communications for V2X scenarios. Rev. Méditerr. Télécommun..

[22] Abboud K., Omar H.A., Zhuang W. (2016). Interworking of DSRC and cellular network technologies for V2X communications: A survey. IEEE Trans. Veh. Technol..

[23] Wang Y., Duan X., Tian D., Chen M., Zhang X. (2016). A DSRC-based vehicular positioning enhancement using a distributed multiple-model Kalman filter. IEEE Access.

[24] Hussain S.M., Yusof K.M., Asuncion R., Hussain S.A., Ahmad A., Sagaya Aurelia S., Hiremath S.S., Subramanian K., Biswas S.K. (2022). An Integrated Approach of 4G LTE and DSRC (IEEE 802.11p) for Internet of Vehicles (IoV) by Using a Novel Cluster-Based Efficient Radio Interface Selection Algorithm to Improve Vehicular Network (VN) Performance. Lecture Notes in Electrical Engineering.

[25] Naik G., Choudhury B., Park J.-M. (2019). IEEE 802.11bd & 5G NR V2X: Evolution of radio access technologies for V2X communications. IEEE Access.

[26] Castañeda Garcia M.H., Molina-Galan A., Boban M., Gozalvez J., Coll-Perales B., Şahin T., Kousaridas A. (2021). A tutorial on 5G NR V2X communications. IEEE Commun. Surv. Tutor..

[27] Shrestha R., Nam S.Y., Bajracharya R., Kim S. (2020). Evolution of V2X Communication and Integration of Blockchain for Security Enhancements. Electronics.

[28] Gebrezgiher Y.T., Jeremiah S.R., Deng X., Park J.H. (2025). Machine learning-based blockchain technology for secure V2X communication: Open challenges and solutions. Sensors.

[29] Barakabitze A.A., Ahmad A., Mijumbi R., Hines A. (2020). 5G network slicing using SDN and NFV: A survey of taxonomy, architectures and future challenges. Comput. Netw..

[30] Wu X., Subramanian S., Guha R., White R.G., Li J., Lu K.W., Bucceri A., Zhang T. (2013). Vehicular communications using DSRC: Challenges, enhancements, and evolution. IEEE J. Sel. Areas Commun..

[31] van den Hoven J. (2008). Information technology, privacy, and the protection of personal data. The Cambridge Handbook of Information and Computer Ethics.

[32] Warren S.D., Brandeis L.D. (1890). The right to privacy. Harv. Law. Rev..

[33] Toll K. (1968). Privacy and freedom. Soc. Work..

[34] Pokhrel S.R., Choi J., Kim H., Kim J. (2020). Towards enabling critical mMTC: A review of URLLC within mMTC. IEEE Access.

[35] Andrews J.G., Buzzi S., Choi W., Hanly S.V., Lozano A., Soong A.C.K., Zhang J.C. (2014). What will 5G be?. IEEE J. Sel. Areas Commun..

[36] Hataba M., Sherif A., Mahmoud M., Abdallah M., Alasmary W. (2022). Security and Privacy Issues in Autonomous Vehicles: A Layer-Based Survey. IEEE Open J. Commun. Soc..

[37] Lu Z., Qu G., Liu Z. (2019). A survey on recent advances in vehicular network security, trust, and privacy. IEEE Trans. Intell. Transp. Syst..

[38] Lee D., Hess D.J. (2022). Public concerns and connected and automated vehicles: Safety, privacy, and data security. Humanit. Soc. Sci. Commun..

[39] Atmaca U.I., Maple C., Dianati M. Emerging privacy challenges and approaches in connected and autonomous vehicle systems. Proceedings of the Living in the Internet of Things (IoT 2019).

[40] Guo Q., Li L., Ban X.J. (2019). Urban traffic signal control with connected and automated vehicles: A survey. Transp. Res. Part C Emerg. Technol..

[41] Elbir A.M., Soner B., Çöleri S., Gündüz D., Bennis M. (2022). Federated learning in vehicular networks. Proceedings of the IEEE International Mediterranean Conference on Communications and Networking (MeditCom), Athens, Greece, 5–8 September 2022.

[42] Moulahi T., Zidi S., Alabdulatif A., Atiquzzaman M. (2021). Comparative performance evaluation of intrusion detection based on machine learning in in-vehicle controller area network bus. IEEE Access.

[43] Aledhari M., Bousselham A., Jazi M.A., Othman M., Ghazi H. (2025). Safeguarding connected autonomous vehicle communication: Protocols, intra- and inter-vehicular attacks and defenses. Comput. Secur..

[44] Pandey M., Pandey S., Kumar A. (2022). Introduction to federated learning. Federated Learning Systems.

[45] Du Z., Zhang Y., Li X., Yang K., Shen X. (2020). Federated learning for vehicular Internet of Things: Recent advances and open issues. IEEE Open J. Comput. Soc..

[46] Liang P.P., Liu T., Zhang K., Yang Q., Shen X. (2020). Think locally, act globally: Federated learning with local and global representations. arXiv.

[47] Chen D., Li X., Wang Y., Huang L., Yu R. (2025). Mobility-aware decentralized federated learning with joint optimization of local iteration and leader selection for vehicular networks. arXiv.

[48] Shaikh F.K., Zeadally S., Exposito E. (2017). Enabling technologies for green Internet of Things. IEEE Syst. J..

[49] Hussain N., Rani P., Chouhan H., Gaur U. (2022). Cyber security and privacy of connected and automated vehicles based on federated learning: Challenges, opportunities, and open issues. Federated Learning Systems.

[50] Fan B., He Z., Wu Y., He J., Chen Y., Jiang L. (2020). Deep Learning Empowered Traffic Offloading in Intelligent Software-Defined Cellular V2X Networks. IEEE Trans. Veh. Technol..

[51] Alladi T., Chamola V., Sahu N., Guizani M. (2020). Applications of blockchain in unmanned aerial vehicles: A review. Veh. Commun..

[52] Alladi T., Chamola V., Sahu N., Venkatesh V., Goyal A., Guizani M. (2022). A comprehensive survey on the applications of blockchain for securing vehicular networks. IEEE Commun. Surv. Tutor..

[53] Vahid S., Tafazolli R., Filo M. (2014). Small Cells for 5G Mobile Networks. Fundamentals of 5G Mobile Networks.

[54] Szabo N. (1997). The Idea of Smart Contracts. https://www.fon.hum.uva.nl/rob/Courses/InformationInSpeech/CDROM/Literature/LOTwinterschool2006/szabo.best.vwh.net/smart_contracts_idea.html.

[55] Gupta M. (2018). Blockchain for Dummies.

[56] Mammen P.M. (2021). Federated learning: Opportunities and challenges. arXiv.

[57] Elgabli A., Park J., Bedi A.S., Bennis M., Aggarwal V. (2020). GADMM: Fast and communication-efficient framework for distributed machine learning. J. Mach. Learn. Res..

[58] Ma C., Zhang J., Zheng Z., Yang K., Shen X., Leung V.C.M. (2020). When federated learning meets blockchain: A new distributed learning paradigm. arXiv.

[59] Joshi G.P., Perumal E., Shankar K., Tariq U., Ahmad T., Ibrahim A. (2020). Toward Blockchain-Enabled Privacy-Preserving Data Transmission in Cluster-Based Vehicular Networks. Electronics.

[60] Guntuka S., Shakshuki E.M., Yasar A., Gharrad H. (2020). Vehicular Data Offloading by Road-Side Units Using Intelligent Software Defined Network. Procedia Comput. Sci..

[61] Zhao O., Liu X., Li X., Singh P., Wu F. (2022). Privacy-preserving data aggregation scheme for edge computing supported vehicular ad hoc networks. Trans. Emerg. Telecommun. Technol..

[62] Wang R., Li H., Liu E. (2021). Blockchain-based federated learning in mobile edge networks with application in Internet of Vehicles. arXiv.

[63] Lugan S., Desbordes P., Brion E., Ramos Tormo L.X., Legay A., Macq B. (2019). Secure architectures implementing trusted coalitions for blockchained distributed learning (TCLearn). IEEE Access.

[64] Geyer R.C., Klein T., Nabi M. (2017). Differentially private federated learning: A client-level perspective. arXiv.

[65] Pokhrel S.R., Choi J. (2020). Federated Learning With Blockchain for Autonomous Vehicles: Analysis and Design Challenges. IEEE Trans. Commun..

[66] Peng Y., Chen Z., Chen Z., Ou W., Han W., Ma J. (2021). BFLP: An adaptive federated learning framework for Internet of Vehicles. Mob. Inf. Syst..

[67] Lu Y., Huang X., Zhang K., Maharjan S., Zhang Y. (2020). Blockchain-empowered asynchronous federated learning for secure data sharing in Internet of Vehicles. IEEE Trans. Veh. Technol..

[68] Phong L.T., Aono Y., Hayashi T., Wang L., Moriai S. (2018). Privacy-preserving deep learning via additively homomorphic encryption. IEEE Trans. Inf. Forensics Secur..

[69] Chen Y., Qin X., Wang J., Yu C., Gao W. (2020). FedHealth: A federated transfer learning framework for wearable healthcare. IEEE Intell. Syst..

[70] Lu Y., Huang X., Dai Y., Maharjan S., Zhang Y. (2019). Blockchain and Federated Learning for Privacy-Preserved Data Sharing in Industrial IoT. IEEE Trans. Ind. Inform..

[71] Verma N., Jain S., Doriya R. (2021). Review on consensus protocols for blockchain. Proceedings of the International Conference on Computing, Communication, and Intelligent Systems (ICCCIS), Greater Noida, India, 19–20 February 2021.

[72] Wang A., Zha Z., Guo Y., Chen S. (2019). Software-Defined Networking Enhanced Edge Computing: A Network-Centric Survey. Proc. IEEE.

[73] Cardona N., Coronado E., Latré S., Riggio R., Marquez-Barja J. (2020). Software-Defined Vehicular Networking: Opportunities and Challenges. IEEE Access.

[74] Chowdhury M., Islam M., Khan Z. (2020). Security of connected and automated vehicles. arXiv.

[75] Hammedi R.A. (2021). Deep learning-based traffic classification in software-defined networking. Proceedings of the 14th IADIS International Conference on Information Systems, Virtual Conference, 3–5 March 2021.

[76] Peng H., Ye Q., Shen X.S. (2019). SDN-based resource management for autonomous vehicular networks: A multi-access edge computing approach. IEEE Wirel. Commun..

[77] Fan Y., Zhang N. (2017). A survey on software-defined vehicular networks. J. Comput. Chem..

[78] Nkenyereye L., Adhi Tama B., Reddy A.G., Song J. (2020). Software-defined vehicular cloud networks: Architecture, applications, and virtual machine migration. Sensors.

[79] Huang C.-M., Chiang M.-S., Dao Duy T., Su W.-L., Xu S., Zhou H. (2018). V2V data offloading for cellular networks based on software-defined networking inside mobile edge computing architecture. IEEE Access.

[80] Azizian M., Cherkaoui S., Senhaji Hafid A. (2017). Vehicle software updates distribution with SDN and cloud computing. IEEE Commun. Mag..

[81] Zhao L., Li J., Al-Dubai A., Zomaya A.Y., Min G., Hawbani A. (2019). Routing schemes in software-defined vehicular networks: Design, open issues, and challenges. IEEE Intell. Transp. Syst. Mag..

[82] Chilès J.-P., Desassis N. (2018). Fifty years of kriging. Geostatistics Valencia 2016.

[83] Zhu M.B., Fan Y., Li H., Li Y., Wang H. (2015). SDN-based routing for efficient message propagation in VANETs. Proceedings of the International Conference on Wireless Algorithms, Systems, and Applications (WASA), Qufu, China, 10–12 August 2015.

[84] Hartenstein H., Laberteaux K. (2010). VANET: Vehicular Applications and Inter-Networking Technologies.

[85] Dong B., Wu W., Yang Z., Li J. (2017). Software Defined Networking Based On-Demand Routing Protocol in Vehicle Ad Hoc Networks. 2016 12th International Conference on Mobile Ad-Hoc and Sensor Networks (MSN).

[86] Zhong H., Wang Y., Li J., Zhang X., Yu R. (2020). A weight-based conditional privacy-preserving authentication scheme in software-defined vehicular network. J. Cloud Comput..

[87] Huang J., Qian Y., Hu R.Q. (2020). Secure and efficient privacy-preserving authentication scheme for 5G software-defined vehicular networks. IEEE Trans. Veh. Technol..

[88] Boualouache A., Soua R., Tang Q., Engel T. (2021). Software-defined location privacy protection for vehicular networks. Proceedings of the International Conference on Information Security Practice and Experience, Nanjing, China, 17–19 December 2021.

[89] Mendiboure L., Chalouf M.A., Krief F. (2020). A scalable blockchain-based approach for authentication and access control in software-defined vehicular networks. Proceedings of the International Conference on Computer Communications and Networks (ICCCN), Honolulu, HI, USA, 3–6 August 2020.

[90] Raja G., Zhang Y., Vasilakos A.V., Lim C., Kim B. (2021). Energy-efficient end-to-end security for software-defined vehicular networks. IEEE Trans. Ind. Inform..

[91] Alsenwi M., Alqaralleh B., Alasmary W., Alsharif M.H., Shuaib K., Alouini M.-S. (2021). Intelligent resource slicing for eMBB and URLLC coexistence in 5G and beyond: A deep reinforcement learning-based approach. IEEE Trans. Wirel. Commun..

[92] Bana A.-S., Rezki Z., Alouini M.-S. (2019). Massive MIMO for Internet of Things connectivity. Phys. Commun..

[93] Sohaib R.M., Kim H., Alsamhi S.H., Guizani M., Ksentini A. (2023). Intelligent resource management for eMBB and URLLC in 5G and beyond wireless networks. IEEE Access.

[94] Zhu X., Jiang S., Wang L., Li H. (2014). Efficient Privacy-Preserving Authentication for Vehicular Ad Hoc Networks. IEEE Trans. Veh. Technol..

[95] Jiang D., Delgrossi L. (2008). IEEE 802.11p: Towards an international standard for wireless access in vehicular environments. Proceedings of IEEE Vehicular Technology Conference (VTC Spring), Singapore, 11–14 May 2008.

